# *Helicobacter pylori* and Respiratory Diseases: 2021 Update

**DOI:** 10.3390/microorganisms9102033

**Published:** 2021-09-26

**Authors:** Marilena Durazzo, Alessandro Adriani, Sharmila Fagoonee, Giorgio Maria Saracco, Rinaldo Pellicano

**Affiliations:** 1 Department of Medical Sciences, University of Turin, C.so A.M. Dogliotti 14, 10126 Turin, Italy; marilena.durazzo@unito.it (M.D.); alexgibs@libero.it (A.A.); giorgiomaria.saracco@unito.it (G.M.S.); 2 Unit of Gastroenterology, Molinette Hospital, Città della Salute e della Scienza, C.so Bramante 88, 10126 Turin, Italy; 3 Institute of Biostructure and Bioimaging, National Research Council, Molecular Biotechnology Center, Via Nizza 52, 10126 Turin, Italy; sharmila.fagoonee@unito.it

**Keywords:** *Helicobacter pylori*, respiratory diseases, asthma, chronic obstructive pulmonary disease

## Abstract

*Helicobacter pylori* (*H. pylori*) is a Gram-negative bacterium involved in the development of gastritis, peptic ulcer disease, gastric adenocarcinoma, and gastric mucosa-associated lymphoid tissue. Unexplained iron deficiency anemia, idiopathic thrombocytopenic purpura and vitamin B12 deficiency have also been related to *H. pylori* infection, whereas for other extra-gastric diseases, the debate is still open. In this review, we evaluate and discuss the potential involvement of *H. pylori* infection in the pathogenesis of several respiratory diseases. A MEDLINE search of all studies published in English from 1965 to 2021 was carried out. Controversial findings have been reported in patients with bronchial asthma, chronic obstructive pulmonary disease, bronchiectasis, lung cancer, tuberculosis, cystic fibrosis, and sarcoidosis. Most of the available literature is concerned with case-control studies based on seroprevalence, with a small sample size and low consideration of confounders, which represents a potential issue. So far, there is no clear evidence of a causal association between *H. pylori* infection and respiratory diseases, and larger studies with appropriate epidemiological design are required.

## 1. Introduction

*Helicobacter pylori* (*H. pylori*) infection is globally widespread, usually acquired during childhood, and often related to low socio-economic class [1]. Although the precise mode of transmission remains unproven, it has been shown that such a microorganism spreads directly from one person to another, mainly by fecal-oral or oral-oral routes [2]. This microaerophilic, Gram-negative bacterium is usually located within the mucus layer of the stomach, and certain ultrastructural details found on its surface (sheathed flagella and urease) are involved in its ability to survive in the surrounding hostile environment [3]. Thus, this disproves the ancient conception of the impossibility for microorganisms to survive in the gastric compartment due to acidity [4]. It is well known that *H. pylori* infection may lead to gastritis, peptic ulcer disease (PUD), gastric adenocarcinoma and gastric mucosa-associated lymphoid tissue (MALT) lymphoma [5], although most infected subjects remain asymptomatic. Furthermore, in the past few years, the possible role of *H. pylori* in many extra-gastric diseases has been investigated [6,7,8]. Among these, accumulating evidence also supports an association with neurodegeneration [9] and nonalcoholic fatty liver disease [6], although some controversy still exists. However, only unexplained iron deficiency anemia, idiopathic thrombocytopenic purpura, and vitamin B12 deficiency have been associated with the latter infection, as reported in the fifth edition of the Maastricht/Florence Consensus Report (2017) [10].

Respiratory diseases represent a leading cause of morbidity and mortality in the world. For this reason, as stated by the World Health Organization (WHO), the prevention, control and cure of these diseases must be a top priority in global decision-making in the health sector [11]. Currently, infections are the leading cause of respiratory diseases in both children and adults, with variable outcomes depending on the causal agent as well as on the host and environmental factors. Since known etiologic agents and risk factors explain the pathogenesis of only a proportion of cases, investigating whether non-traditional agents have a causal role in the pathogenetic steps of respiratory diseases is of primary importance.

Early epidemiologic studies on the relationship between *H. pylori* infection and respiratory diseases have been supported by the findings on animal models showing that the presence of the microorganisms in the gastric compartment could be associated with lung injury, as indicated by the increased expression of inflammatory mediators and markers of endothelial dysfunction [12]. Over time, a series of publications, mainly reporting the findings of epidemiologic studies, has focused on this issue and has provided controversial results.

The aim of this narrative review is to evaluate the consistent data available regarding a potential involvement of *H. pylori* infection in the pathogenesis of respiratory diseases.

## 2. Materials and Methods

**Figure 1:**
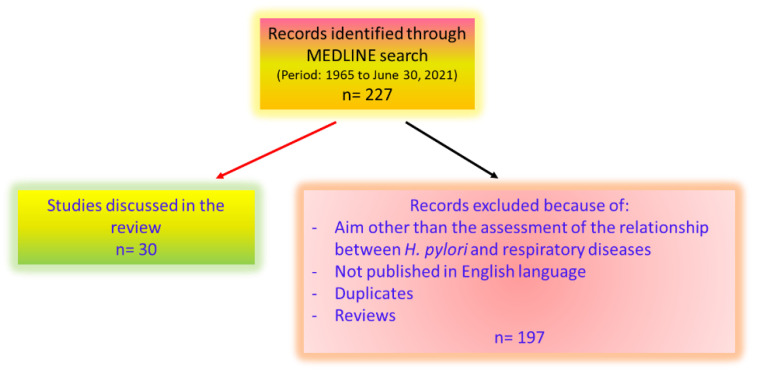
Flow chart of included studies.

A MEDLINE search of all published studies from 1965 to 30 June 2021 was carried out in order to identify all appropriate publications (Figure 1). The following medical subject headings were used: *Helicobacter pylori*, *Helicobacter*, asthma, bronchitis, chronic obstructive pulmonary disease or COPD, bronchiectasis, lung cancer, tuberculosis, cystic fibrosis, and sarcoidosis. Systematic reviews and meta-analyses were used to summarize the evidence, if available.

## 3. Results

### 3.1. H. pylori Infection and Asthma

Bronchial asthma is a chronic inflammatory disease of the airways, characterized by airflow obstruction and the associated presence of intermittent symptoms, including wheezing, dyspnea, chest tightness, and coughing. The prevalence of asthma has increased markedly in developed countries in recent years [13]. From a pathophysiological point of view, exposure to defined allergens or to various non-specific stimuli initiates a cascade of cellular activation events resulting in acute and chronic inflammatory processes mediated by a complex assortment of locally released cytokines and other mediators. This leads to altered airway smooth muscle responsiveness, mucus hypersecretion and damaged epithelium. 

Considering that several studies did not show a causal relationship, but often, an inverse association between *H. pylori* infection and allergic asthma [14,15], a protective effect of this microorganism against allergic diseases including asthma, especially in children and young people, was hypothesized around fifteen years ago (the so-called “hygiene hypothesis”). The authors associated the reduction of H. pylori prevalence with the rise in asthma cases as well as other allergic disorders in children, assuming a possible relationship [16]. Over time, multiple epidemiological studies were performed on this topic and a recent meta-analysis, including 18 observational studies with 17,196 enrolled children, reported a significant negative association between *H. pylori* and the risk for childhood asthma (odds ratio [OR] = 0.68; 95% confidence interval [CI]: 0.54–0.87; *p* = 0.002), particularly in those harboring the more virulent strains (according to *cytotoxin-associated gene A* [*CagA*] status) (OR = 0.58; 95% CI: 0.35–0.96; *p* = 0.034). No significant difference among studies regarding participant age, geographical region, study design and diagnostic method for *H. pylori* detection was observed [17]. In recent years, the association between asthma or other allergic diseases and *H. pylori* has been intensively investigated. In a case-control study including more than 10,000 patients, *H. pylori* infection was found in 31%, asthma in 10.4%, and allergic rhinitis in 16% of them, without any significant association; however, in patients with abdominal obesity, *H. pylori* infection was associated with 30-40% reduced OR of asthma and 25% reduced OR of allergic disorders [18]. Moreover, in a case-control study performed in Greece including 27 pediatric patients with asthma and 54 controls, an inverse association between *H. pylori* and asthma was confirmed (OR = 0.1; 95% CI: 0.039–0.305; *p* = 0.026) [19]. In a cohort study, 16% of children who were uninfected at 2 and 10 years of age developed asthma at 16 years vs. none of the children with *H. pylori* infection at 2 years of age [20] (Table 1).

**Figure 2:**
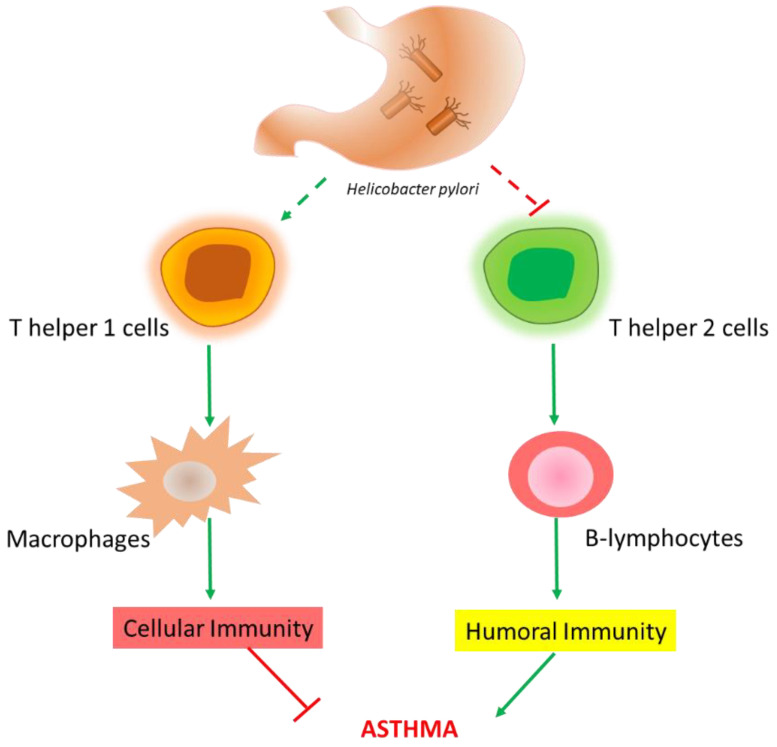
Proposed pathway (Th1 activation and Th2 inhibition) explaining the protective effect of *H. pylori* infection against asthma (hygiene hypothesis).

A possible explanation for this inverse relationship between *H. pylori* and asthma, according to the “hygiene hypothesis”, is the possibility that infectious agents can inhibit allergic T helper (Th) 2 cells pathways, thus eliciting a Th1-type immune response. Furthermore, the neutrophil-activating protein of *H. pylori* (HP-NAP) increases interferon (IFN)-γ production and decreases interleukin (IL)-4, thus driving Th1 inflammation and inhibiting Th2 responses (Figure 2) [21]. Another possibility involves the inverse correlation between *H. pylori* infection and gastroesophageal reflux disease (GERD), as the latter can worsen asthma and this is a risk factor for developing GERD [22,23]. 

Nevertheless, some authors consider *H. pylori* only a marker of poor household hygiene. This stems from the evidence from studies testing the hypothesis of a protective effect in relation to asthma in populations with poor hygiene and low *H. pylori* prevalence (for example in Malaysia and Indonesia), which did not confirm this effect [24]. Furthermore, the results of studies and consequently meta-analyses could be affected by the diagnostic method used to diagnose *H. pylori* infection (mainly serology that does not discriminate between current and past infection).

### 3.2. H. pylori Infection and Chronic Obstructive Pulmonary Disease

Chronic obstructive pulmonary disease (COPD) is a chronic inflammatory lung disease characterized by the presence of chronic bronchitis or emphysema that may lead to the development of airflow limitation. Acute infections, especially respiratory viral infections, and/or exposure to pollutants are the main causes of COPD. Nevertheless, other factors may contribute to COPD, such as the exacerbation of other respiratory diseases and non-respiratory diseases (e.g., heart failure, thromboembolism) [25]. Whatever the cause, the principal pathological features of chronic bronchitis are inflammation of airways and hypertrophy of mucus glands, with increased mucus secretion and airway obstruction.

In a Danish study, Rosenstock et al. assessed the association of *H. pylori* infection with lifestyle, chronic disease, body indices, and age at menarche in 2913 adults. The authors found a higher prevalence of chronic bronchitis in women with *H. pylori* seropositivity compared to uninfected women (OR = 1.6; 95% CI: 1.1–2.5) [26]. Other studies reported higher rates of *H. pylori* seropositivity in patients with COPD or chronic bronchitis [27,28,29,30,31] but these were all case-controlled in design and included limited sample sizes. In a recently published case-control study, Bener et al. reported the prevalence of *H. pylori* seropositivity in 529 patients with type 2 diabetes mellitus and in controls. The authors found that the former group had a significantly higher prevalence of *H. pylori* compared to controls. Furthermore, in diabetic patients, seropositivity was significantly associated with chronic bronchitis (OR = 2.09; 95% CI: 1.16–3.77) [32]. In a retrospective cohort study using data from the Taiwanese National Health Insurance Research Database and including 5941 adults with a new diagnosis of *H. pylori*, the overall hazard ratio of COPD was 1.84 (95% CI = 1.57–2.17), compared to the non-*H. pylori* cohort, after adjusting for age, sex, and comorbidities [33]. On the other hand, a study performed in Korea (a country with a high prevalence of *H. pylori* infection) showed no differences in seroprevalence of *H. pylori* between COPD patients and controls (45.8% in 201 COPD vs. 52.2% in 402 controls, *p* = 0.134). Furthermore, there were no significant differences in the rate of lung function decline (annual forced expiratory volume in 1 s or forced vital capacity [FVC]) in subjects with COPD and airflow limitation [34]. Similarly, in a retrospective study conducted in a health screening population among 3619 subjects, 1849 (51.1%) tested positive for *H. pylori* immunoglobulin (Ig)G and, in the first year, 95 (2.6%) patients were diagnosed with COPD, without any difference in seropositivity for *H. pylori* compared to non-COPD subjects (*p* = 0.756). There was no significant difference in the incidence of COPD between the seronegative (2.2%) and the seropositive group (2.0%; *p* = 0.728), and in the decline rates of the mean FVC and forced expiratory volume in the first second (FEV_1_) (35.38 vs. 34.34 mL/year for FVC, *p* = 0.389; 39.23 vs. 37.49 mL/year for FEV1, *p* = 0.086). Moreover, the decline in lung function or COPD progression was not influenced by *H. pylori* eradication [35]. Bennett et al. assessed in 67 lung transplant patients whether *H. pylori* seropositivity was associated with their outcome. While the overall prevalence of *H. pylori* infection in this cohort was similar to that reported in the general population, it was lower among patients with COPD and higher among those with pulmonary fibrosis. No correlation was found between *H. pylori* infection and graft outcome, either in patients with pulmonary fibrosis or in those with COPD. Nevertheless, more patients who required three or more post-transplant re-hospitalizations belonged to the *H. pylori* seropositive group (*p* = 0.046) [36].

**Table 1:** Summary of the cumulative results of the main studies included.

Respiratory Disease		Result	Reference	Publication Year
	Meta-analysis of 18 studies	Negative association with *H. pylori* infection	[16]	2021
ASTHMA	Case-control study including 10,000 patients	Only in obese patients *H. pylori* associated with 30–40% OR of asthma ↓	[17]	2019
	Cohort study	16% of children uninfected at 2 and 10 years developed asthma at 16 years versus none of the children with *H. pylori* at 2 years	[19]	2020
CHRONIC OBSTRUCTIVE PULMONARY DISEASE (COPD)	Case-control study including patients with type 2 diabetes mellitus and controls	In diabetics, seropositivity was significantly associated with chronic bronchitis	[31]	2020
	Retrospective cohort study (Taiwan)	Significant association between *H. pylori* and COPD	[32]	2017
	Case-control study (Korea, country with high burden of *H. pylori*)	No association between *H. pylori* and COPD	[33]	2016

Samareh Fekri et al., in a cross-sectional study, evaluated the relationship between *H. pylori* infection and COPD through several approaches: serologic testing for *H. pylori* IgG, real-time polymerase chain reaction (PCR) of bronchoalveolar lavage (BAL) and trans-bronchial biopsy urease tests. Sixty adults with COPD were included. The prevalence rate of *H. pylori* infection was 10% according to real time PCR, 88.3% according to the serology test and 0% based on the urease test. The relationship between *H. pylori* presence (based on PCR) and disease severity and prognosis was not statistically significant. These findings supported the hypothesis of direct injury and chronic inflammation via inhalation and aspiration resulting in *H. pylori* colonization [37].

Thus, the role of *H. pylori* infection on the development and progression of COPD remains unclear.

### 3.3. H. pylori Infection and Bronchiectasis

Bronchiectasis is an abnormal and permanent dilatation of the bronchi or their branches, due to acute or chronic inflammation, and destruction of the elastic and muscular tissue of the bronchial wall. This disease may be caused by cystic fibrosis, past severe infections, genetic diseases like α-1 antitrypsin, allergic aspergillosis or immune system diseases. Sometimes, the cause remains unexplained, configuring the picture of idiopathic disease [38,39].

In this context, it has been hypothesized that the inhalation of *H. pylori* or its endotoxins into the respiratory tract could lead to a chronic activation of inflammatory mediators. In 1998, Tsang et al. first described a significantly higher serum IgG positivity against *H. pylori* in 100 consecutive patients with bronchiectasis compared to healthy controls (76.0% vs. 54.3% respectively, *p* = 0.001) [40]. The following year, Tsang et al. reported a higher *H. pylori* CagA+ seroprevalence in bronchiectasis patients than in controls (24% vs. 11.7%, *p* = 0.03) [41]. However, Angrill et al. were unable to detect *H. pylori* by histochemical and immunochemical staining in bronchial tissue from patients with bronchiectasis and positive serology [42]. Similarly, Gülhan et al. did not find evidence of *H. pylori* DNA in either BAL fluid or in lung tissues by using PCR, from patients with bronchiectasis. In addition, they did not find a statistically significant difference in anti-*H. pylori* IgG level between patients and controls [43]. Furthermore, in another study, there were no significant differences between bronchiectasis patients and controls regarding *H. pylori* positivity in BAL fluid, gastric juice, and urea breath test [44].

### 3.4. H. pylori Infection and Lung Cancer

Lung cancer represents a major health problem and is the leading cause of cancer deaths worldwide. It is mostly caused by inhalation of environmental carcinogens, but 10–15% of cases in Western countries and up to 25% of cases in Asia are diagnosed in never-smoker subjects [45].

The potential role of chronic infections and inflammation in the stages of carcinogenesis have been widely investigated for many years, but evidence on the relationship between *H. pylori* infection and risk of lung cancer is still controversial, with a limited number of underpowered studies reporting contrasting results. Two case-control studies, conducted by Philippou et al. [46] and Najafizadeh et al. [47], showed no significant association between *H. pylori* infection and lung cancer. Another case-control study compared 700 lung cancer patients (350 lung adenocarcinoma cases and 350 squamous cell carcinoma cases) with 700 controls, showing no significant association between *H. pylori* seropositivity and adenocarcinoma (OR = 1.1, 95% CI: 0.75–1.6) or squamous cell carcinoma (OR = 1.1, 95% CI: 0.77–1.7). Similar results were reported for CagA− and CagA+ *H. pylori* seropositivity [48]. On the other hand, other case-control studies revealed significantly higher rates of seropositivity for antibodies against *H. pylori* in patients with lung cancer than in controls [49,50,51]. A meta-analysis, published in 2013, included the above-mentioned studies and calculated the pooled OR [52]. A significantly increased risk of lung cancer in patients with *H. pylori* infection (pooled OR = 2.29, 95% CI: 1.34–3.91, *p* = 0.01) was reported. The authors, thus, concluded that the results should be interpreted cautiously due to the inclusion of underpowered studies.

### 3.5. H. pylori Infection and Tuberculosis

Tuberculosis (TB) is a chronic infectious disease sustained by the bacterium *Mycobacterium tuberculosis* (*M. tuberculosis*), which generally affects the lungs but can also involve other organs. Current estimates are that about one-third of the world population is infected by *M. tuberculosis.* Nevertheless, infected people without active disease are considered latent TB cases [53].

In 1992, Mitchell et al. reported that a previous history of active pulmonary TB might be associated with *H. pylori* infection in southern China [54]. Subsequently, Filippou et al. assessed the seroprevalence of *H. pylori* in a cohort of 80 TB patients before starting anti-tuberculosis therapy. These authors observed a seroprevalence of 87.5% in the TB group compared to 61.4% among controls (*p* < 0.004) [55]. Sanaka et al. showed opposite results in a serology-based case-control study. No significant difference in *H. pylori* seroprevalence was found when comparing 40 inpatients treated with anti-tuberculosis chemotherapy for no more than 3 months, 43 TB patients on chemotherapy for more than 3 months, and 60 controls (73.3%, 65% and 69.8%, respectively) [56]. However, a potential confounding factor could be represented by the possible eradication of *H. pylori* by anti-tuberculosis drugs, such as rifampicin, streptomycin and rifabutin [57,58]. Furthermore, published studies included mainly patients with active TB, who usually represent a small proportion (about 20%) of those with *M. tuberculosis* infection. Thus, any potential association might have been masked.

Bustamante-Rengifo et al. proposed two hypothetical models to explain the possible interaction between *H. pylori* and *M. tuberculosis.* In the first one, the *H. pylori* vacuolating cytotoxin that interferes with the maturation of dendritic cells and antigen-presenting cells as well as inhibits the proliferation of T lymphocytes could be involved. This compromises CD4^+^ T cell-activation which could affect susceptibility to acquiring *M. tuberculosis.* In the second model, regulatory T (Treg) cells induced by *H. pylori* could downregulate Th1 and Th17 responses, thus affecting host immunity against *M. tuberculosis* [53]. Increased predisposition to *H. pylori* infection and enhanced mycobacterial survival and replication, hypothesized in TB patients, may be sustained by the same HLA-DQ serotype, hence inducing susceptibility to both bacteria [59,60].

### 3.6. H. pylori Infection and Cystic Fibrosis

Cystic fibrosis is a progressive inherited multisystem disease that affects primarily the lungs and the digestive system. It is known that in this disease, mutations in the cystic fibrosis transmembrane conductance regulator (CFTR) are associated with dysfunction in CFTR protein. This leads to changes in mucus that becomes thick and sticky in various organs.

Few studies have examined a possible correlation between *H. pylori* and cystic fibrosis. Drzymała-Czyż et al. assessed the prevalence of *H. pylori* infection, using breath tests with isotope-labeled urea in 79 cystic fibrosis patients compared to 302 healthy controls, but no significant difference was found [61]. In a study by Yahav et al., the authors found a lower prevalence of *H. pylori* infection in cystic fibrosis patients than in non-cystic fibrosis controls (16.6% and 30.0%, respectively), assessed by using specific monoclonal antibodies for fecal *H. pylori* antigen. However, due to the small number of cystic fibrosis patients enrolled (*n* = 30) in this study, the difference was not statistically significant [62]. A seroprevalence study, conducted by Israel et al., included 70 cystic fibrosis patients. The authors reported an initial seropositivity rate of 47% (33/70) for *H. pylori* IgG antibody, but after pre-adsorption of these sera with *Pseudomonas* proteins, a marked decrease in *H. pylori* seropositivity (8%, 6/70) was observed, highlighting a cross-reactivity between *H. pylori* antigens and *Pseudomonas* antibodies [63].

Interesting findings arose from basic research. Wen et al. reported a relationship between *H. pylori* infection and CFTR downregulation in human duodenal epithelial cells. The authors found that this event occurred through an increase in transforming growth factor-β production. The latter induced the phosphorylation of p38 mitogen-activated protein kinase [64]. This link between *H. pylori* infection and CFTR downregulation has triggered scientific interest in understanding whether the microorganism could be involved in some step of the pathogenesis of cystic fibrosis-induced gastrointestinal and extra-gastrointestinal damage and whether its eradication could reverse this CFTR expression status.

### 3.7. H. pylori Infection and Sarcoidosis

Sarcoidosis is an inflammatory disease characterized by the development and growth of granulomas that affects, in most cases, the lungs and lymph glands. Gastrointestinal involvement of sarcoidosis is rare but when it occurs, gastric localization is the most common effect [65]. The cause of sarcoidosis is unknown but it has been hypothesized that a ubiquitous trigger (chemical, infectious or environmental agent) in genetically susceptible individuals could induce an altered immune response leading to the disease [66].

There are only few reports regarding a possible association between *H. pylori* and sarcoidosis, without any clear causal relationship. Herndon et al. found an increased incidence of specific *H. pylori* and urease IgG antibodies in sarcoidosis patients compared to controls [67]. Koyama et al. reported the presence of *H. pylori* in a patient with idiopathic granulomatous gastritis [68], although the granulomatous lesion took several months to heal after bacterial eradication. In another case report, a subject with symptomatic gastric sarcoidosis was misdiagnosed as PUD and confounded by *H. pylori* infection [69]. So far, the evidence of an etiopathogenetic relationship between the bacterium and gastric or extra-gastric sarcoidosis is missing.

## 4. Discussion

In recent years, several studies have evaluated the possible relationship between *H. pylori* infection and various respiratory disorders, such as bronchial asthma, COPD, bronchiectasis, lung cancer, TB, cystic fibrosis and sarcoidosis. For many of these, a causal relationship has not been confirmed, primarily due to some important sources of heterogeneity: the limitations due to epidemiological design of the studies, the low consideration of confounding variables, lack of appropriate controls and the methods used to assess *H. pylori* infection.

Considering study designs, most of the available literature is based on case-control studies (which could never prove a causal relationship) with small sample sizes (and consequent increased risk of type II error). Only prospective studies provide a direct estimate of the risk of developing disease in subjects with a certain characteristic relative to those without that characteristic. None of the studies investigating the relationship between *H. pylori* infection and respiratory diseases in humans is prospective or based on the criteria proposed to consider a causal role for an agent, i.e., temporality or presence of the cause before onset of the disease [70]. However, as *H. pylori* infection is acquired during childhood and lasts for decades [5], and since most of the respiratory diseases have a late onset in life, it can be objected that *H. pylori* infection is most likely to precede the occurrence of the disease.

Confounders (either in cases or in the control population) represent another possible influencing variable. For example, low socio-economic status is related to both *H. pylori* infection [71] and risk of developing chronic bronchitis [72], but as the bacterium is acquired during childhood, matching for socio-economic status should be performed during childhood and not at the time of the study. Tobacco use represents another possible confounder: it is well known that the prevalence of COPD [72] and lung cancer [73] is higher in smokers, whereas variable prevalence of *H. pylori* infection in smokers has been reported. Thus, the possible impact of cigarette smoking on this type of respiratory diseases and *H. pylori* infection should be regarded as a potential study limitation.

The methods used to diagnose *H. pylori* infection can be classified as invasive or non-invasive, the former being based on biopsy specimens obtained at endoscopy. The choice of the test depends on the clinical context. These methods vary in sensitivity and specificity, which may lead to misclassification biases. Endoscopic-based tests have a sensitivity depending on the number of bioptic samples obtained during endoscopy, the gastric site of collection, and the consideration of taking drugs that could influence the location of the bacterium in the stomach [74]. Among non-invasive tests, urea breath tests and stool antigen tests both have high sensitivity and specificity (above 90%), whereas serologic testing for *H. pylori* IgG has a specificity of less than 80% for active *H. pylori* infection [5,75] due to antibodies persistence for years. For this reason, the presence of these antibodies should be considered a marker of exposure rather than a marker of ongoing infection, limiting their use in clinical practice due to the risk of treating patients without previous exposure. Nevertheless, as mentioned above, most of the available literature on the relationship between respiratory manifestations and *H. pylori* infection is based on seroprevalence studies that are considered appropriate from an epidemiological point of view.

**Figure 3:**
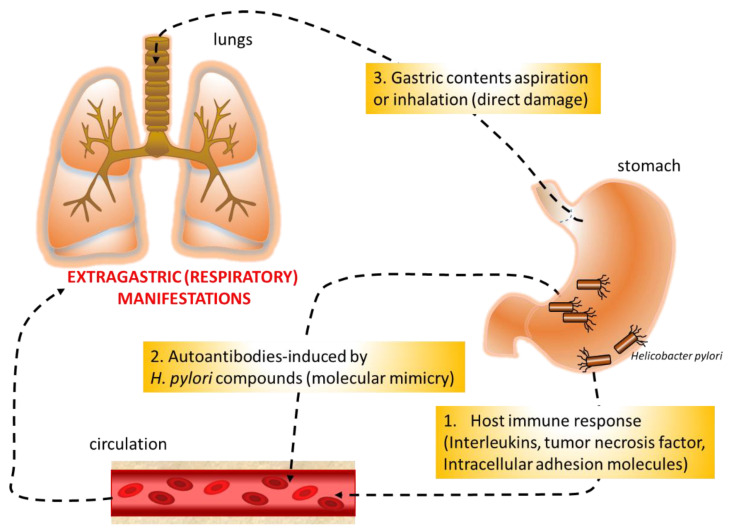
The three hypotheses to explain the potential relationship between *Helicobacter pylori* and respiratory diseases.

The mechanisms that could correlate respiratory diseases with *H. pylori* infection are still unclear, but several hypotheses have been formulated (Figure 3): host immune response to the bacterium that could induce gastric and extra-gastric tissue damage, molecular mimicry that can result in the cross-activation of autoreactive T or B cells by bacterial components, and direct damage with chronic airway inflammation by bacterium aspiration or inhalation. *H. pylori* stimulates humoral, adaptive, local, and systemic immune response. The potential involvement of the microorganism in the pathogenesis of extra-gastroduodenal diseases via systemic immune response is supported by the evidence that the long-term inflammation generated by *H. pylori* might raise cytokine levels (IL-1, IL-4, IL-8, IL-10 and tumor necrosis factor-α) in the bloodstream [76]. Furthermore, studies involving animal models have shown that this increase occurred not only in serum but also in the lung tissue of infected mice, together with an increase in metalloproteinases and markers of endothelial dysfunction such as intracellular adhesion molecule-1 and intracellular adhesion molecule-5, involved in leukocyte adhesion and migration into the sub-endothelium. On the contrary, lungs from non-infected animals did not show these alterations [12]. This could reflect the genetic modification occurring in the lung as a result of gastric *H. pylori* infection. In particular, in mice, it has been shown that while in the absence of a local inducing agent, genes classically involved in pathogen recognition (*Smad3* and *Smad5*) were not affected in the lung, and in the case of gastric *H. pylori* infection, the expression of these genes was downregulated in the stomach and increased in the lung. As a consequence, expression of the SMAD protein plays a crucial role in Th cell differentiation [77]. The autoimmune mechanism, based on molecular mimicry between *H. pylori* lipopolysaccharides and the host tissues, has been demonstrated in the stomach and could also be involved in extra-gastrointestinal manifestations associated with this infection. The hypothesis of the induction by H. pylori of anti-self reactions was proposed after antibodies with reactivity to the gastric mucosa were detected in the sera of infected patients [78,79,80,81].

Some consistent results showed a possible negative association between *H. pylori* and risk for childhood asthma, particularly in the case of more virulent strains [17]. The “hygiene hypothesis”, according to which infectious diseases can inhibit allergic Th2 pathways, thus eliciting a Th1 immune response, has been proposed [21]. However, as discussed above, studies conducted in populations with poor hygiene and low *H. pylori* prevalence did not support this model. Finally, the possible inverse correlation between *H. pylori* infection and GERD has also been supposed, as this condition can worsen asthma, and the latter is a risk factor for developing GERD [22,23]. However, none of these possible mechanisms, or other aspects, such as genetic predisposition and the impact of *H. pylori* eradication on asthma incidence, have been studied in detail. 

An intriguing field of interest involves both the potential role of the airway’s microbiota in the host’s local immune regulation and their contribution in the pathogenesis of respiratory tract diseases [82]. In this context, the potential influence of this microbial community on the systemic consequences of *H. pylori* infection remains to be elucidated. 

A limitation of our study is the exclusive use of MEDLINE database for conducting our literature search. This database, which is the primary component of PubMed, is a great resource for medical research because it contains more than 30 million references to journal articles. Furthermore, MEDLINE is accessed for free and permits the visualization of online early versions before print publication by various journals [83]. The associated use of Web of Science and Scopus databases would have allowed a more complete collection of data. However, since this is a narrative review, there is less of a risk of influencing the results compared to a systematic review.

## 5. Conclusions

The question as to whether *H. pylori* is an innocent bystander, a protective agent or a trigger of respiratory diseases cannot yet be answered. On the other hand, these are often multifaceted disorders, the mechanism of which cannot be explained by only one cause. Hence, the need for larger studies with appropriate epidemiological design to investigate a potential causal relationship between *H. pylori* infection and respiratory diseases is evident.

## Data Availability

https://pubmed.ncbi.nlm.nih.gov/ (30 June 2021).
